# Stem cell-derived kidney organoids: engineering the vasculature

**DOI:** 10.1007/s00018-019-03401-0

**Published:** 2019-12-05

**Authors:** Marije Koning, Cathelijne W. van den Berg, Ton J. Rabelink

**Affiliations:** 1 Department of Internal Medicine-Nephrology, Leiden University Medical Center, Leiden, The Netherlands; 2 Einthoven Laboratory of Vascular and Regenerative Medicine, Leiden University Medical Center, Leiden, The Netherlands

**Keywords:** Transplantation, Angiogenesis, Vasculogenesis, Endothelial cells, Nephrons

## Abstract

Kidney organoids can be generated from human pluripotent stem cells (PSCs) using protocols that resemble the embryonic development of the kidney. The renal structures thus generated offer great potential for disease modeling, drug screening, and possibly future therapeutic application. At the same time, use of these PSC-derived organoids is hampered by lack of maturation and off-target differentiation. Here, we review the main protocols for the generation of kidney organoids from human-induced PSCs, discussing their advantages and limitations. In particular, we will focus on the vascularization of the kidney organoids, which appears to be one of the critical factors to achieve maturation and functionality of the organoids.

## Introduction

Chronic kidney disease (CKD) is highly prevalent worldwide and increases the risk of cardiovascular and all-cause mortality [1–3]. Treatment options are limited, and once patients progress to end stage renal disease (ESRD), they become dependent on renal replacement therapy, requiring either dialysis or kidney transplantation. Dialysis is a strenuous treatment associated with high morbidity and mortality. Kidney transplantation is currently the best available treatment for ESRD, but suffers from a shortage of donor kidneys and necessitates the lifelong use of immunosuppressive drugs.

The publication of protocols to generate 3D kidney organoids from human-induced pluripotent stem cells (hiPSCs) was, therefore, met with great enthusiasm. Human iPSCs can be obtained by reprogramming adult cells from any individual, either healthy or diseased, offering unprecedented possibilities for treatment and disease modeling [4]. Over the past 5 years, several protocols have been developed to differentiate human PSCs to kidney organoids [5–8]. All are based on the development of the kidney in an embryo. Here, the nephron including the glomerulus, proximal and distal tubules, loop of Henle, and connecting tubule is derived from the metanephric mesenchyme (MM) [9–11], whereas the collecting duct and ureter are derived from the ureteric bud (UB). Inductive interaction between these two progenitor populations is essential for correct development and 3D organization of the kidney [12, 13].

Protocols for iPSC-derived kidney organoids generally yield nephron structures containing glomerular, proximal tubular, and distal tubular structures that, in many cases, are surrounded by stromal and endothelial cells [5–8, 14]. In addition, some authors claim the generation of collecting duct like structures [7, 14].

Kidney organoids have already been used to study kidney development [5, 6, 15], model certain (early onset) kidney diseases [8, 16–19] and screen for tubular nephrotoxicity [6–8]. However, before they can be applied for regenerative medicine purposes, there are still many limitations to overcome. Kidney organoids frequently contain off-target cell populations [20–23], suffer from interexperimental variability [21], display limited 3D organization, contain far too few nephrons to replace renal function, and, unless they are generated from a patient’s own cells, are likely to elicit an immune response upon transplantation [24, 25]. Another great concern is that, although kidney organoids frequently contain some endothelial cells, they lack a functional vasculature.

Kidneys are highly vascularized organs whose correct development, functionality, and maintenance require the presence of blood flow and the interaction with vascular endothelial cells. In embryology, glomerular podocytes and mesangial cells develop in close conjunction with endothelial cells and their maturation as well as the development of the glomerular filtration barrier are disturbed when endothelial cells are lacking [26–28]. In adult kidneys, specialized endothelial cells in the glomerulus, peritubular capillaries and ascending and descending vasa recta contribute to glomerular filtration, tubular urine concentrating ability, and interstitial fluid drainage [29]. Consequently, the applicability of kidney organoids lacking a perfused vascular network is limited.

It has been shown that transplantation of kidney organoids in mice results in vascularization by perfused blood vessels as well as significant maturation, confirming the benefit of a functional vasculature for progressive organoid development [30–32]. Very recently, researchers aimed to achieve similar results in vitro by culturing kidney organoids with flow over their top surface. They observed an increase in the number of endothelial cells, which formed networks that, in some cases, invaded organoid glomerular structures. However, they were unable to convincingly show that the vascular networks were fully perfusable [33]. In addition, for both the transplantation studies and the in vitro culture under flow, it is unclear to what degree the blood vessels that are formed possess the properties of the specialized renal vasculature in vivo.

Here, we review the main protocols to generate 3D kidney organoids from human PSCs and discuss the importance of a functional vasculature in kidney organoids. We compare the process of kidney vascularization in embryology to that occurring after transplantation of PSC-derived organoids to murine hosts and upon culturing them under flow in vitro. Finally, we consider the potential and limitations of in vivo and in vitro vascularized kidney organoids.

## Human pluripotent stem cell-derived kidney organoids

To differentiate iPSCs to a specific tissue type, detailed knowledge about its embryonic development is indispensable. The protocols for the generation of iPSC-derived kidney organoids are, therefore, based on extensive research in mammalian nephrogenesis and their establishment was accompanied by important developmental discoveries.

In embryology, the metanephric kidneys are derived from the intermediate mesoderm (IM), which gives rise to both the MM and the UB [34, 35]. In response to glial-derived neurotrophic factor (GDNF) produced by the MM, the UB protrudes from the nephric duct into the MM and branches to form the collecting duct system [36–38]. The MM cells surrounding the UB tips condense to form the cap mesenchyme (CM), which contains the progenitor cells for the entire nephron [9–11].

Dependent on canonical Wnt signaling activated by UB-derived Wnt9b, the CM forms a pretubular aggregate (PTA) [39, 40]. The PTA expresses Wnt4, which induces mesenchyme-to-epithelial transition (MET) through non-canonical signaling and gives rise to the renal vesicle (RV) [41, 42]. Recently, it was discovered that the formation of the RV occurs gradually over time with the recruitment of nephron progenitor cells (NPCs) from the CM into the RV. The timing of recruitment plays an important role in the proximal–distal patterning of the nephron. NPCs that are recruited first form the distal tubular precursors and are located closest to the ureteric bud tip, the second recruits form the proximal tubular precursors, and the last cells to enter the RV give rise to podocyte precursors [43]. The RV subsequently develops into the comma-shaped body, S-shaped body, capillary loop stage, and, finally, the mature nephron, which is connected to the UB-derived collecting duct system.

### Protocols for human iPSC-derived kidney organoids

Since renal organogenesis depends on the interaction between the MM and UB, induction of both progenitor populations will be essential to eventually generate complete kidneys from iPSCs. Researchers, therefore, set out to determine their precise developmental origin. Although it was known that both progenitor populations derive from the IM, the processes responsible for the differentiation towards either the UB or the MM lineage had not been elucidated.

To investigate this, Taguchi et al. extensively studied the development of NPCs in mice, starting at the MM stage and moving backwards to the PS stage step by step. Possible precursor populations were identified, isolated, and tested for their capacity to differentiate to the next stage in vitro. This enabled them to test their hypotheses as well as differentiation conditions for mouse embryonic nephron precursors.

It was discovered that the presence of metanephric nephron progenitors was restricted to the posterior IM (PIM), with cells in the anterior IM (AIM) contributing to mesonephric nephrons and the ureteric bud. Additional experiments demonstrated that the PIM is derived from caudal Brachyury positive (T+) primitive streak (PS) cells that remain posteriorized until E8.5, while the AIM cells migrate anteriorly in an earlier stage [5].

The obtained knowledge was used by Taguchi et al. and several other research groups to develop protocols for the differentiation of human PSCs to 3D kidney organoids [5–8].

**Fig. 1:**
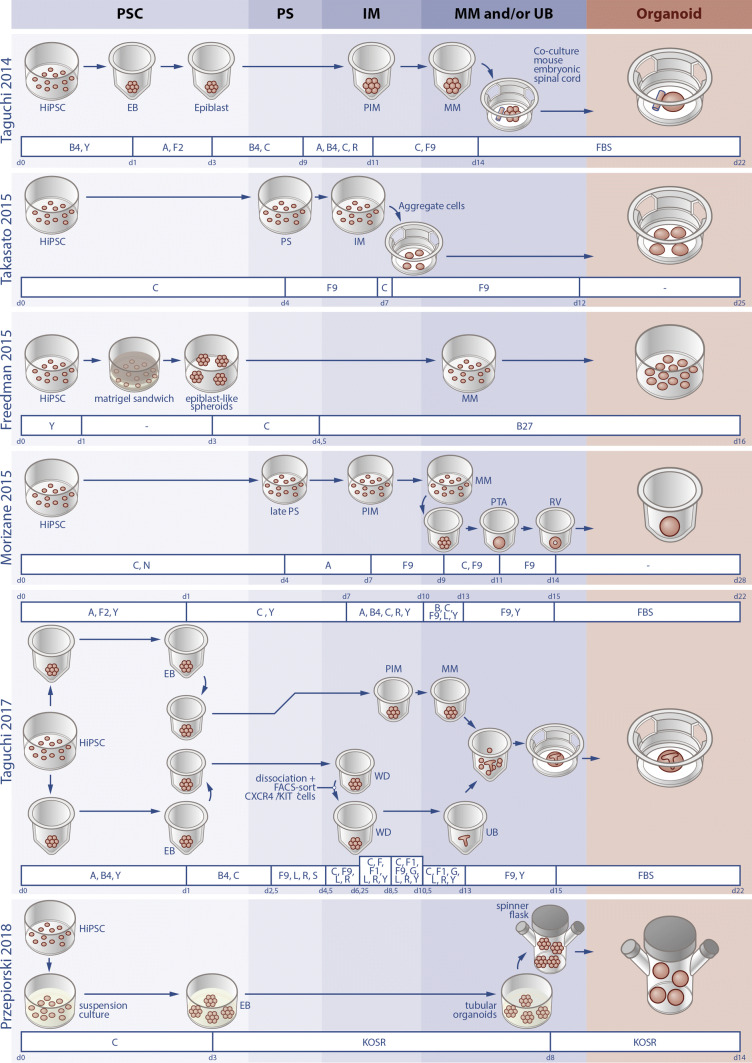
Overview of protocols for the generation of kidney organoids from human iPSCs. The schematically shown protocols are described in the main text and include the co-culture of MM with mouse embryonic spinal cord or separately induced UB, culturing on an air–liquid interface, using a Matrigel sandwich, induction of nephron progenitor cells followed by renal vesicle formation, and suspension culture. *A* activin A, *B* BMS493, *B4* bone morphogenetic protein 4, *B27* B27 medium, *C* CHIR 99021, *EB* embryoid body, *F* fibroblast growth factor, *FBS* fetal bovine serum, *G* glial cell-derived neurotrophic factor, *hiPSC* human-induced pluripotent stem cells, *IM* intermediate mesoderm, *KOSR* knockout serum replacement, *L* LDN193189, *MM* metanephric mesenchyme, *N* noggin, *PIM* posterior intermediate mesoderm, *PS* primitive streak, *PSC* pluripotent stem cells, *PTA* pretubular aggregate, *R* retinoic acid, *RV* renal vesicle, *S* SB431542, *UB* ureteric bud, *WD* wolffian duct, *Y* Y27632

The protocol by Taguchi et al. entails the generation of embryoid bodies, followed by mesoderm induction with BMP4 and CHIR (Wnt agonist), stimulation of the obtained mesoderm with Activin A, BMP4, CHIR, and retinoic acid to achieve differentiation to PIM and, finally, addition of CHIR and FGF9 to generate MM cells. Co-culture of the hiPSC-derived MM with mouse embryonic spinal cord results in the formation of 3D kidney organoids containing nephron-like structures [5] (Fig. 1).

Morizane et al. [6] developed a chemically defined protocol for the generation of NPCs and kidney organoids. They tested varying doses and duration of CHIR treatment and growth factors to determine the optimal conditions for the induction of late PS and PIM. By treating hiPSCs with CHIR and Noggin for 4 days to obtain PS, followed by stimulation with Activin A for 3 days, they generate PIM cells with 80–90% efficiency. Subsequent addition of FGF9 to the PIM cells is sufficient to achieve differentiation to MM within 2 days. The obtained MM cells are replated in 96-well round bottom low attachment plates and the formation of renal vesicles is induced by treatment with CHIR and FGF9 during 2 days, followed by 3 days with FGF9 only. From day 14 of differentiation onwards, no growth factors are added to the media and kidney organoids with nephron-like structures form through self-organization (Fig. 1).

Freedman et al. cultured hiPSCs between two layers of Matrigel to induce the formation of cavitated spheroids that maintain pluripotency. By treating these spheroids with CHIR for 1.5 days, differentiation to mesenchyme is induced, which subsequently undergoes MET upon culture in B27-supplemented media, resulting in the formation of organoids with nephron-like structures [8] (Fig. 1).

Takasato et al. hypothesized that, since the AIM is formed by cells that migrate anteriorly from the PS early on, these are exposed to the strong Wnt signaling in the PS for a shorter period of time than those that migrate in a later stage to form the PIM. Therefore, it might be possible to induce both the AIM and the PIM from PSCs by optimizing the duration of CHIR treatment prior to replacement by FGF9 [7]. They tested this theory by treating PSCs with CHIR for 3, 4, or 5 days and checking for the expression of AIM/UB (LHX1, GATA3) and PIM/MM (HOXD11, EYA1) markers at days 7 and 18 of differentiation using qPCR (day 7) and immunofluorescent stainings (days 7 and 18). Based on these experiments, they claimed that exposing hPSCs to CHIR for 4 days followed by FGF9 for 3 days induces both PIM and AIM simultaneously. To subsequently generate organoids, the cells were dissociated, reaggregated to form cell pellets, and cultured on transwell filters where they were treated with a CHIR pulse followed by FGF9 for 5 more days. Afterwards, growth factors were removed and it was reported that over the next 6–13 days, 3D kidney organoids containing nephron-like structures as well as collecting duct networks formed [7] (Fig. 1).

Collecting ducts were defined by the authors as tubular structures that co-expressed GATA3 and ECAD. However, these markers are not specific for the collecting duct only, and have been shown to be expressed in the MM-derived distal tubules and connecting tubules as well [44]. In addition, the collecting duct tree in vivo is a highly organized branching structure that connects the tubular system to the calyces and ureter, ensuring a single urinary exit path. The structures termed collecting ducts in the in vitro kidney organoids did not display this morphology. Also, single-cell RNA sequencing analysis of the organoids failed to identify a clear ureteric bud and/or collecting duct cell population [20, 23]. These limitations have led to discussion in the field about the nature of the GATA3+, ECAD+ structures in the kidney organoids. Future research aimed specifically at unravelling the identity of these cells will hopefully clarify this.

Despite the differences in culture conditions between the protocols discussed above, the resulting organoids as reported by the authors show many similarities. In all cases, nephron-like structures containing podocytes (WT1/NPHS1+/PODXL+), proximal tubules (LTL+/CUBN+/LRP2+), and distal tubules (ECAD+) were demonstrated through immunofluorescent stainings and confocal microscopy [5–8]. Some, but not all, authors also described the presence of endothelial cells (CD31+, /vWF+) [7, 8], stromal cells, ‘early mesangial cells’ [7], and/or epithelial cells expressing a marker specific for the ascending loop of Henle (UMOD+) [6, 7]. Bowman’s capsule like structures were demonstrated in several of the previously discussed papers [6–8] and it was shown later on that these consist of parietal epithelial cells (CLDN1+, PAX8+) [22] that display aberrant morphology in organoids generated from PAX2 knockout iPSCs [45]. However, it is unclear whether this variation in reported cell types represents true differences between the obtained organoids. The methods used to characterize the organoids were not identical and reports of direct comparisons between protocols are scarce. One study compared organoids generated through the Takasato and Morizane protocol using single-cell RNA sequencing (scRNAseq), and demonstrated that these were very similar with regard to the cell populations present, although differences in the fractions of these populations were detected [20]. Of note, this and other scRNAseq studies have demonstrated that kidney organoids do not consist solely of renal cell types, but also contain off-target stromal, neural, and/or muscle cell populations [20–22, 46].

### Three-dimensional organization of kidney organoids

An important question regarding the organoids generated through all the above protocols is to what degree their 3D organization resembles that in the in vivo kidney. The in vivo kidney is a complex structure consisting of many different cell types which are meticulously organized. In organoids, glomerular, proximal tubular and distal tubular structures have been reported to connect in the right order [5–8], but this is not always the case and the lack of a branching collecting duct system precludes the development of a morphologically correct structure with full corticomedullary organization. This is also illustrated by the ‘Loop of Henle’ cells in the organoids. Although these cells express uromodulin, a marker specific for epithelial cells in the ascending Loop of Henle, they have not been shown to form the elongated U-shaped structure that in vivo extends deep into the medulla before looping back to the renal cortex. In this area, there is ample room for improvement.

Last year, Taguchi et al. [14] developed a new protocol with the aim of improving the 3D organization of kidney organoids by generating a branching collecting duct system. The authors utilized a method reminiscent of the dissociation–reaggregation strategies applied for primary fetal tissue-derived kidney organoids [47, 48]. Instead of aggregating MM and UB tissue that was isolated from murine embryos, they used separately induced mouse ESC-derived MM and UB. For the generation of MM, they employed an adapted version of their earlier protocol [5]. The protocol for the induction of UB was newly developed based on the developmental processes involved in the differentiation to the UB lineage in mice, which the authors studied using a backward approach. As discussed previously, UB progenitors migrate anteriorly from the T + PS stage earlier on than MM progenitors and are, therefore, exposed to Wnt signaling for a shorter period of time. However, the authors found that solely reducing the duration of CHIR treatment was insufficient to induce UB progenitors and developed a complex protocol that includes a step in which cells are dissociated and FACS-sorted for the Wolffian duct (WD) progenitor markers CXCR4+/KIT+, to obtain PSC-derived UB [14]. Upon combination of separately induced mouse ESC-derived MM and UB with primary mouse embryonic stromal progenitors, kidney organoids containing nephrons connected to an arborized collecting duct were formed [14]. Unfortunately, when the protocol was adapted for human iPSCs, it was much less successful. The obtained UB displayed only minimal branching capacity when cultured in Matrigel, and upon combination with hiPSC-derived MM, branching morphogenesis did not occur, nor did the formation of nephrons from the MM.

This might be due to the lack of stromal progenitors, a suboptimal differentiation of either the MM or the UB, or a problem with the reaggregation assay [14].

### Scaling up organoid production

All of the previously discussed protocols for kidney organoid differentiation are labor intensive, often requiring manual pipetting and/or aggregation steps. This limits the efficiency and reproducibility of organoid generation, both of which are essential to apply them for drug and nephrotoxicity screening or renal replacement therapy. Recently, several strategies aimed at improving these aspects were published.

Przepiorski et al. showed that the laboriousness and cost of kidney organoid generation can be reduced using suspension culture in BPEL supplemented with CHIR followed by DMEM with KnockOut Serum Replacement (KOSR). To further scale up production, the organoids can be transferred to spinner flasks on day 8 of differentiation [49] (Fig. 1). The organoids generated seem similar to those obtained with earlier protocols based on immunofluorescent stainings, but a direct comparison was not performed. This straightforward protocol is inexpensive due to the use of BPEL and the replacement of FGF9 or B27 by KOSR. However, the use of BPEL, which contains bovine serum albumin, makes the protocol susceptible to batch variation and might reduce the reproducibility of the organoids.

Czerniecki et al. took a different approach to increasing the efficiency of kidney organoid generation and focused on their suitability for high-throughput screening (HTS). To this end, the previously discussed Matrigel-sandwich protocol [8] was adapted to adherent culture in 96- or 384-well plates [22]. In each well, many very small organoids containing about five nephron-like structures per organoid appeared, surrounded by areas with non renal cells, indicating that the protocol is not fully efficient in inducing differentiation to the kidney lineage. However, as the authors demonstrated, culturing in 384-well plates facilitates efficient testing of differentiation conditions and toxicity screening.

In summary, various protocols have been developed to generate kidney organoids containing nephron-like structures. Important limitations of these organoids are their limited 3D organization, the presence of off-target cell populations, and the lack of a functional vasculature and of a single urinary exit tract.

However, in their current form, the organoids have already been shown to be suitable to model certain kidney diseases.

## Using PSC-derived kidney organoids for disease modeling

Over the past few years, the first studies reporting the use of 3D hPSC-derived kidney organoids for disease modeling were published. Generally, two main approaches are employed to obtain diseased organoids: inducing mutations/knockouts in healthy hPSC lines using CRISPR/Cas9 or generating hiPSCs from patients. In both cases, it is of great importance to distinguish differences between affected and healthy organoids caused by the disease from those due to line to line variability. HPSC lines from different donors can exhibit marked variation in differentiation potential [50, 51] and kidney organoids generated from different cell lines or even in a different batch from the same cell line vary in composition and maturation [21]. To reduce the influence of line to line variability, organoids derived from diseased cell lines should be compared to those from isogenic controls, and ideally, multiple cell lines should be investigated.

Freedman et al. focussed on polycystic kidney disease (PKD) and generated PKD1 and PKD2 knockout PSCs from a human ESC line using CRISPR/Cas9 [8, 19]. Upon differentiation to kidney organoids using their adherent protocol, about 7% of the organoids formed fluid filled cysts [8, 19]. This percentage rose to 75% when they removed the organoids from the surrounding stroma on day 21 of differentiation and subsequently cultured them in suspension, indicating a role for adherent forces in preventing tubular cyst formation. The high number of cysts in the PKD organoids was not due solely to the adapted culture conditions, as only 5% of organoids from isogenic control hPSCs developed cysts under the same circumstances [19].

In addition to the PKD1 and PKD2 knockout PSCs, the authors differentiated cell lines from two patients with autosomal dominant PKD (ADPKD) and one with autosomal recessive PKD (ARPKD) to kidney organoids. Due to great variability in the capacity to differentiate as well as the propensity to form cysts in these patient cell lines, they were not used for additional experiments [19].

The PKD knockout model can be useful to study the process of early (prenatal) cyst formation and to perform drug screening. However, the immaturity of tubules in kidney organoids, the lack of a collecting duct, and the scarcity of stromal tissue in this model might limit its applicability for modeling postnatal PKD, in which the majority of cysts arise from distal nephron segments and collecting ducts and extensive fibrosis is a characteristic feature. Also, it cannot be used for personalized medicine purposes, as it does not reflect the variable phenotype of PKD in patients. This would require the optimization of kidney organoid culture from patient iPSC lines.

Forbes et al. generated an uncorrected and a gene-corrected hiPSC line from a patient with nephronophthisis-related ciliopathy (NPHP-RC) due to compound–heterozygous mutations in IFT140. After differentiation of both cell lines to kidney organoids, they analyzed the morphology of the cilia through manual, blinded scoring of individual cilia. In organoids derived from the uncorrected iPSCs, they found that 59% of the cilia displayed the clubbed morphology that was previously described in IFT140 null mice, compared to 12% in the gene-corrected organoids [17]. In addition, they performed differential gene expression analysis, which indicated downregulation of genes involved in apicobasal polarity in epithelial cells derived from patient organoids. Spheroid culture of these epithelial cells after isolation from the organoids was impaired accordingly [17].

However, studying disease-related defects in apicobasal polarity in intact kidney organoids will be very challenging as their immature tubular epithelium is characterized by incomplete polarization [30]. More advanced modeling of NPHP-RC in kidney organoids will, therefore, require tubular maturation.

Kim et al. and Freedman et al. investigated the effect of the knockout of podocalyxin, which leads to early postnatal death in mice, on the podocytes in iPSC-derived kidney organoids [8, 15]. They demonstrated that the formation of microvilli by these podocytes was greatly reduced compared to isogenic controls, with the podocytes clustering closer together and forming lateral cell–cell junctions. Subsequent analysis of podocytes in prenatal PODXL−/− mice revealed a similar phenotype [15]. Although podocalyxin mutations in patients with kidney disease are uncommon, these results demonstrate the suitability of kidney organoids to study developmental processes.

Recently, two different research groups used kidney organoids to study congenital nephrotic syndrome (CNS) [16, 18]. Tanigawa et al. investigated early podocyte abnormalities in CNS due to an NPHS1 missense mutation [16, 18]. They differentiated patient-derived iPSCs to kidney organoids using a modified version of the Taguchi 2017 protocol and detected a difference in NEPHRIN localization when comparing these to organoids derived from a gene-corrected control cell line [16]. Similarly, Hale et al. [18] used iPSCs derived from a CNS patient with compound heterozygous NPHS1 mutations to generate kidney organoids and observed reduced levels of NEPHRIN and PODOCIN protein compared to healthy controls. Of note, the control cell line used in this study was not isogenic.

Unfortunately, slit diaphragm formation, which is impaired in patients with NPHS1 mutations, could not be studied in organoids in vitro, since the slit diaphragm does not develop under these conditions.

These studies demonstrate that in vitro kidney organoids are suitable to study certain genetic tubular and (early onset) podocyte diseases. However, there are some general limitations to consider. First, CRISPR/Cas9 was used by most authors to either induce or correct disease-causing mutations. Although this is a very potent technology, it is important to realize that it can also have off-target effects, which might influence results [52–54]. Second, in all studies using patient-derived diseased cell lines, these were generated from a single patient and thus a single genetic background. Considering the previously discussed line to line variability of hPSCS, replicating the findings with a cell line from a different background would enhance the strength of the results. Third, beside the PKD organoids, the phenotypes demonstrated are mainly those of the early stages of disease. This is likely due to the immature character and the lack of a functional vasculature of the organoids. Unfortunately, this limits their applicability. For instance, diseases affecting the GBM or slit diaphragms cannot be modeled, since these do not develop in organoids in vitro. Diabetic nephropathy, in which injury of the glomerular endothelial cells plays an important role, cannot be fully recapitulated in their absence, and the modeling of tubular channelopathies would require the maturation of the involved electrolyte channels and transporters, which has not yet been shown. Also, more advanced disease modeling and drug screening with functional read-outs would require glomerular filtration and urine production by the organoids, which is not possible without a functional vasculature.

We, therefore, believe that the vascularization of kidney organoids will be an important step to increase their potential and will focus on this aspect in the remainder of this review.

## Vascularization is essential for kidney functionality and development

Mature mammalian kidneys receive approximately 20–25% of cardiac output. Renal blood flow is essential for virtually all kidney functions, including but not limited to glomerular filtration, blood pressure regulation, and acid–base and electrolyte balance.

Each human kidney is supplied with blood through the renal artery that, at the site of the renal hilum, branches into segmental arteries which give rise to the interlobar arteries travelling towards the cortex. Around the border between the cortex and medulla, the interlobar arteries form the arcuate arteries, from which the interlobular arteries branch towards the periphery of the kidney [55]. There, they give rise to the afferent arterioles which are lined with continuous endothelium and, at the glomerular hilum, contain juxtaglomerular cells that produce and secrete renin in response to blood pressure changes. The afferent arteriole branches to form the glomerular capillary network. The highly fenestrated endothelial cells in this network are part of the glomerular filtration barrier and contribute to its development and maintenance through paracrine cross-talk with podocytes and mesangial cells [26, 27, 56]. The glomerular capillaries drain into the efferent arteriole which, in the case of cortical glomeruli, supplies the cortical peritubular capillary network that enables reabsorption of fluid and electrolytes through endothelial cells with diaphragmed fenestrae [57]. In juxtamedullary glomeruli, the efferent arterioles form the medullary microcirculation by giving rise to the descending vasa recta (DVR), which are lined with continuous endothelium and descend into the medulla in vascular bundles. In the inner medulla, they drain into the diaphragmed fenestrated ascending vasa recta (AVR) [55, 58] which possess lymphatic like qualities that are essential for fluid drainage from the medullary interstitium [29]. Together, the DVR and AVR play an important role in urinary concentrating ability through the countercurrent exchange mechanism.

In kidney development, the importance of vascularization becomes apparent early on and has been demonstrated most extensively for the glomerulus. Glomerular vascularization begins once the renal vesicle has developed through the comma- into the S-shaped body stage, with the development of a vascular cleft between the podocyte precursors and proximal tubular segment. Endothelial progenitor cells expressing VEGFR-2 (Flk1) enter the vascular cleft dependent on VEGFA produced by immature podocytes [59, 60]. They join to form capillary loops which subsequently develop a lumen through TGF-β1-mediated apoptosis of superfluous endothelial cells [61]. The capillary loops branch to form the capillary tuft and the endothelial cells develop fenestrae. These are initially, like in the peritubular capillaries, bridged by diaphragms, but lose these as the cells mature into specialized glomerular endothelial cells with undiaphragmed fenestrae [62, 63].

Disturbance of the glomerular vascularization process can have severe consequences. In mice, heterozygosity for podocyte-specific VEGF-A leads to glomerular endotheliosis and severe proteinuria, whereas a complete absence of podocyte-derived VEGF-A results in glomeruli lacking endothelial cells, disabling the development of a functional filtration barrier, and perinatal death [26]. Antibody-mediated blockade of VEGF activity in newborn mice, in which nephrogenesis is still ongoing, leads to the development of many glomerular structures lacking capillaries [64]. Postnatally, deletion of VEGFR-2 leads to a significant damage to glomerular endothelial cells [65]. These severe phenotypes demonstrate the importance of podocyte-to-endothelial cell signaling for glomerular endothelial cell recruitment as well as survival. Conversely, endothelial cells are essential for podocyte maturation. In the absence of endothelial cells, podocytes do develop but retain an immature phenotype, with effacement of foot processes and lack of slit diaphragms [26].

Signaling between mesangial cells, the third major cell type in the glomerulus, and endothelial cells also plays a pivotal role in glomerular development. PDGFRβ-positive mesangial cells enter the glomerulus in response to PDGFβ produced by endothelial cells [27]. Mesangial cells, in turn, contribute to the looping of glomerular capillaries, which fails to occur in the absence of mesangial cells [66, 67].

Therefore, cross-talk between endothelial cells, podocytes, and mesangial cells is indispensable for the development, maturation, and maintenance of functional glomeruli.

Unfortunately, none of the previously discussed protocols for kidney organoid generation produce organoids containing a functional vasculature. Some iPSC-derived organoids do contain endothelial cells, but these are not organized into blood vessels and do not enter the glomerulus [7, 8, 30]. Addition of vascular endothelial growth factor (VEGF) during organoid differentiation increases the number of endothelial cells, but fails to induce invasion of the glomerulus [22].

The process of embryonic kidney vascularization can provide important clues for the generation kidney organoids with a perfusable vascular network.

## Kidney vascularization in embryonic development: vasculogenesis or angiogenesis?

The development of the human embryonic vasculature starts with the formation of a primitive vascular plexus by differentiation of mesoderm-derived angioblasts, a process called vasculogenesis. Subsequently, this network is expanded and remodeled through angiogenesis; the formation of blood vessels from the existing vasculature by sprouting and intussusception [68, 69].

The process responsible for kidney vascularization and the origin of renal endothelial cells are still debated. Possibilities are vasculogenesis by in situ differentiation of angioblasts that either arise from progenitors in the metanephros or migrate into it from the systemic circulation, angiogenesis from pre-existent systemically connected blood vessels or a combination of both.

Most studies exploring the mechanisms of embryonic kidney vascularization are based on murine models. Support for vasculogenesis came from studies demonstrating the presence of endogenous endothelial progenitor cells within mouse embryonic kidneys. In E10 mouse metanephric kidneys, Flk1+ angioblasts [70] and Tie1 expressing cells [71] unconnected to the systemic vasculature were found and a lineage tracing study in mice showed that a portion of the peritubular capillary endothelium is derived from Foxd1-positive renal stromal cells [72].

More elaborate support for vasculogenesis comes from transplantation studies in which embryonic kidneys were transplanted into murine hosts, followed by the analysis of the origin of the blood vessels inside the graft. Although the results of these experiments were variable, in most cases, at least a part of the glomerular vasculature was graft-derived, indicating a role for vasculogenesis [71, 73, 74] (Table 1). A limitation of these studies is the difficulty in establishing the origin of the donor-derived endothelial cells. Although their presence supports the vasculogenesis theory, it does not provide solid proof, since either endothelial progenitor cells or mature endothelial cells may have entered the metanephric kidney through angiogenesis prior to transplantation. Researchers tried to preclude this possibility by transplanting avascular grafts, but since blood vessels are present within the metanephric kidney from E11, this is nearly impossible.

**Table 1:** Studies reporting the origin of endothelial cells in blood vessels supplying the graft after transplantation of embryonic metanephroi, embryonic organoids, or PSC-derived kidney organoids to a murine host

Author	Journal	Year	Graft	Host	Graft location	Duration Tx	Origin ECs in vascularized graft
Hyink	Am J Physiol	1996	Metanephroi mice E11–12	Mice	Anterior eye chamber	6 days	Small vessels predominantly donor Large vessels predominantly host
Robert	Am J Physiol	1996	Metanephroi mice E12	Mice Neonatal Adult	Neonatal mice Kidney capsule Adult mice Kidney capsule Anterior eye chamber	6–7 days	Neonatal host: combination of host and donor Adult host: Kidney capsule: mostly donor Anterior eye chamber: small vessels donor, large vessels host
Loughna	Angiogenesis	1997	Metanephroi mice E11/E12	Mice—neonatal	Nephrogenic renal cortex	7–10 days	Predominantly donor
Rogers	Am J Physiol Regul Integr Comp Physio	2001	Metanephroi rats E15	Mice—immunosuppressed, uninephrectomized	Omentum	2 weeks	At least partly host (did not stain for donor EC)
Takeda	Transplant Immunology	2006	Metanephroi pigs week 4	Rats—immunosuppressed	Omentum	5, 7 or 8 weeks	Host
Dekel	Nature Medicine	2003	Metanephroi Human: week 7–8 Pig: week 4	Mice—immunodeficient	Kidney capsule	4 weeks	At least partly host (did not stain for donor ECs)
Xinaris	JASN	2012	Embryonic kidney organoids (mice E11.5)	Rats—athymic nude, uninephrectomized	Kidney capsule	3 weeks	Donor
Sharmin	JASN	2016	hiPSC-derived kidney organoids	Mice—immunodeficient	Kidney capsule	20 days	Host
Bantounas	Stem Cell Reports	2018	hESC-derived kidney organoids	Mice—SCID/beige	Subcutaneous	12 weeks	At least partly donor (did not stain for host ECs)
Van den Berg	Stem Cell Reports	2018	hESC and hiPSC-derived kidney organoids	Mice—NOD/SCID	Kidney capsule	7–28 days	Combination of host and donor
Tran	Developmental Cell	2019	hESC-derived kidney organoids	Mice—NOD/SCID	Kidney capsule	10 days	Host

Evidence for angiogenesis in embryonic kidney vascularization is convincing. In the transplantation studies discussed above, the larger vasculature was host-derived in almost all cases [73, 75–77] (Table 1). Furthermore, a recent elaborate spatiotemporal analysis of kidney vascularization in mice indicated such a large role for angiogenesis that the authors proposed an angiogenesis-only mechanism [78]. In this study, high-resolution confocal imaging of normally developed whole metanephric mouse kidneys was performed from the start of metanephric kidney development at E10.5 until birth. At E10.5, CD31+ capillaries were detected neighbouring the UB and the avascular MM was surrounded by scattered CD31+ endothelial cells. Next, the capillaries formed a vascular ring around the UB stalk and a dense network between the Wolffian duct and the MM. By E11.5, vessels from this dense capillary network had entered the MM, and by E12.5, the entire MM was vascularized. Further analysis showed that from E11 onwards, all blood vessels in the metanephric kidney were connected to the embryonic circulation and contained erythroid cells [78].

Although this meticulously performed study provides strong support for angiogenesis, it does not rule out a contribution of vasculogenesis. The scattered CD31+ endothelial cells surrounding the MM at E10.5 may have formed through vasculogenesis and the erythroid cells present in the metanephric vasculature through hemovasculogenesis.

The elaborate research discussed above has provided strong evidence for angiogenesis and largely ruled out a vasculogenesis-only mechanism, although a contribution of vasculogenesis cannot be rejected.

**Fig. 2:**
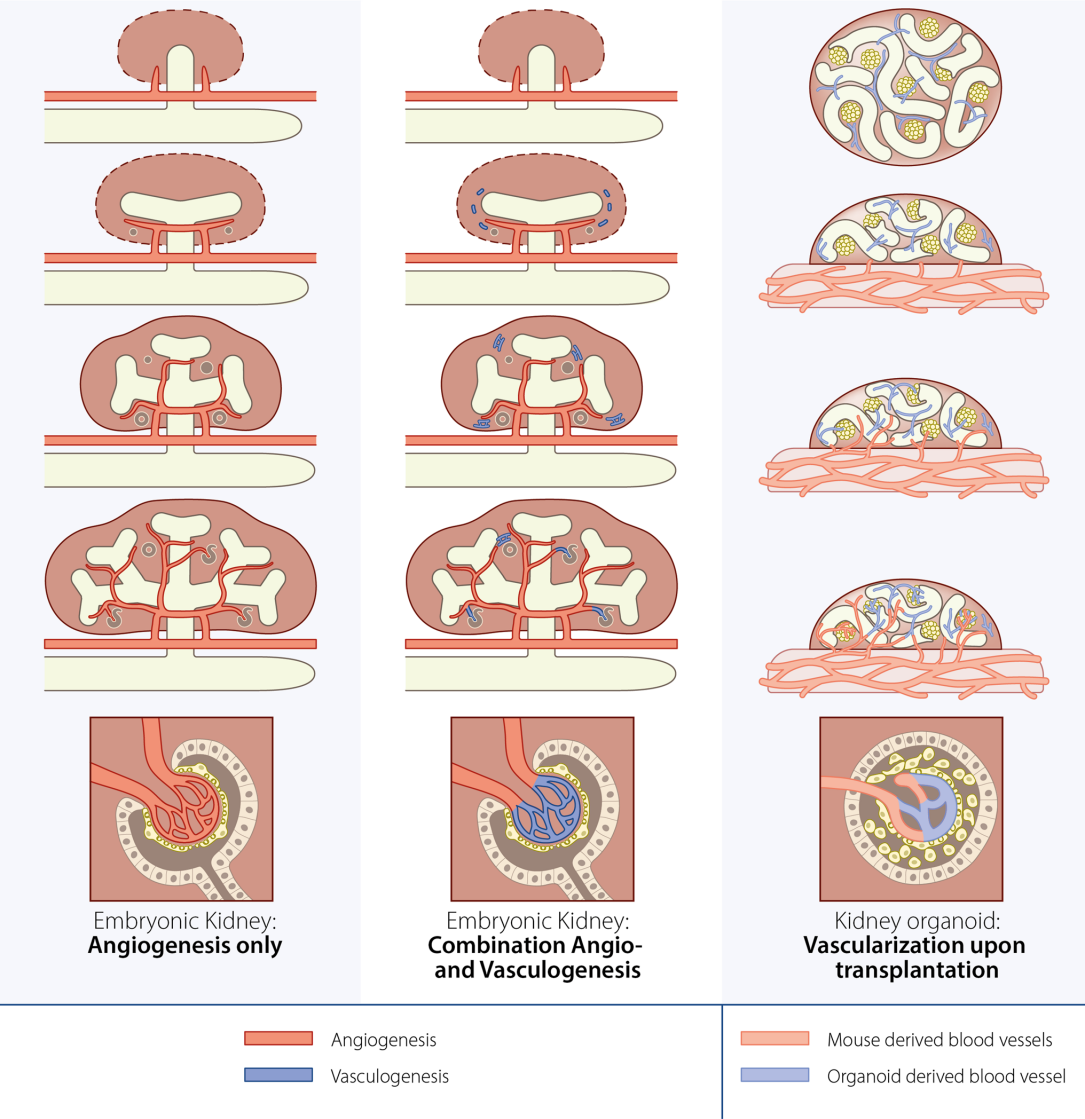
Schematic representation of possible mechanisms of kidney vascularization in embryonic development versus hPSC-derived kidney organoid vascularization upon transplantation. Left and middle: The ureteric bud (white) protrudes from the nephric duct into the metanephric mesenchyme (dark red) and branches over time to form the collecting duct system. Left: angiogenesis only hypothesis. The metanephric kidney is vascularized exclusively through angiogenesis by blood vessels connected to the systemic vasculature (red). Middle: vasculogenesis-only hypothesis. The metanephric kidney is vascularized through a combination of angiogenic vessels (red) and vessels formed through vasculogenesis (blue) from endothelial progenitor cells (blue) present in the metanephric mesenchyme. Right: vascularization of hPSC-derived kidney organoids upon transplantation under the kidney capsule of a murine host. Prior to transplantation, the organoid (dark red circle) contains glomerular structures (clusters of yellow podocytes), tubular structures (white tubes) and endothelial cells (light blue). Upon transplantation, host-derived blood vessels (light red) invade the organoid, and connect to some of the organoid-derived endothelial cells. The organoid is vascularized by a combination of host- and donor-derived endothelial cells. This schematic representation is not based on quantification studies

Therefore, we believe the embryonic development of the intricate vascular network of the kidney depends on either a combination of angio- and vasculogenesis or on angiogenesis only (Fig. 2).

## Vascularization and maturation of kidney organoids in in vivo and in vitro models

Considering the role of angiogenesis in the vascularization of embryonic metanephroi, it seemed likely that vascularizing kidney organoids would require co-culture with blood vessels possessing angiogenic potential. This is challenging to establish in vitro, but in vivo models are available. As discussed previously, transplantation of human, murine or pig metanephroi to the omentum, anterior eye chamber or under the kidney capsule of murine hosts leads to vascularization of the graft [71, 73–77, 79, 80]. Subsequently, the transplanted metanephroi grow and differentiate [75, 77, 79–83], developing mature looking nephrons that function to a certain degree [77, 79, 81–83].

### In vivo vascularization of kidney organoids derived from embryonic mouse tissue

Researchers hypothesized that the transplantation of embryonic organoids generated through dissociation and reaggregation of E11.5 mouse kidneys would yield similar results. However, upon transplantation under the kidney capsule of rats for 3 weeks, vascularization was minimal and maturation of tubular but not of glomerular structures occurred [84]. This seemed to be due to insufficient VEGF production by the graft, as pre- and post-treatment with this growth factor markedly improved both vascularization and maturation. Inside the transplanted organoids, identified by a staining for mouse-specific anti-CENP-A, the authors demonstrated the presence of peritubular and glomerular capillaries lined by donor-derived endothelium (MECA32+, RECA-1−) and containing red blood cells. Transmission electron microscopy (TEM) imaging of glomerular structures demonstrated that glomerular endothelial cells were not fully mature and frequently lacked fenestrations. Podocytes were in varying stages of maturation and in some areas, a glomerular basement membrane (GBM) appeared between endothelial cells and podocytes [84]. The presence of mesangial cells was not convincingly shown. Although it is difficult to distinguish between host- and donor-derived cells in TEM images, it is unlikely that these glomerular structures were derived from the adult rat host, considering their immature features.

The authors further showed that the transplanted organoids achieved a certain level of functionality by injecting FITC-conjugated BSA and FITC-labeled dextran into the host vasculature prior to fixation. Both were found to colocalize with megalin in proximal tubular structures, suggesting that glomerular filtration and tubular endocytosis had occurred [84].

Although these results are promising, the requirement of external VEGF administration to obtain them implies that the intrinsic capability to produce VEGF is reduced in these embryonic organoids compared to metanephric kidneys. This might be due to the relatively small proportion of VEGF producing podocyte precursors in the in vitro organoids; although these were not quantified by the authors, the number of cells stained for the early podocyte marker WT-1 seems limited in the published image and nephrin positive cells were not detected.

In a different study, similar outcomes were observed upon transplantation of ‘organ buds’, consisting of dissociated E13.5 mouse embryonic kidney cells, human umbilical vein endothelial cells (HUVECs), and mesenchymal stem cells (MSCs) inside the cranium of immunodeficient mice. The organ buds were quickly vascularized and the injection of Lucifer Yellow combined with intravital microscopy revealed glomerular perfusion and filtration [85].

### In vivo vascularization of hPSC-derived kidney organoids

In 2016, Sharmin et al. [31] transplanted hiPSC-derived kidney organoids generated using the Taguchi 2014 protocol under the renal capsule of immunodeficient mice. Two rods soaked in VEGF and aggregates of HUVECs and MSCs were transplanted along with the organoids. 20 days after transplantation, the glomeruli in the organoids were vascularized by host-derived mouse ECs and showed signs of maturation, with the formation of foot processes, slit diaphragm-like structures, a double layered glomerular basement membrane (GBM), and fenestrated endothelial cells [31]. The effect of transplantation on the tubular structures was not described. Although VEGF or aggregates of HUVECs and MSCs were co-transplanted in most experiments, they were omitted in a few cases. This did not hamper vascularization, raising doubts concerning the necessity of adding these factors when transplanting hiPSC-derived kidney organoids [31].

Indeed, in 2018, it was shown that hPSC-derived kidney organoids are capable of producing VEGF themselves [30] and two papers were published simultaneously describing their successful vascularization and improved maturation upon transplantation in immunodeficient mice without external VEGF administration [30, 32].

Van den Berg et al. [30] differentiated hiPSCs and hESCs to kidney organoids using an adapted version of the Takasato 2015 protocol and transplanted these under the renal capsule of mice. After 7–28 days, vascularization and functional perfusion of the organoids by a combination of host- and graft-derived blood vessels were demonstrated using live in vivo imaging through an abdominal imaging window (Fig. 2). In addition, transmission electron microscopy (TEM) analysis showed progressive maturation of glomerular and tubular structures over time. The level of maturity varied between structures, but overall improved markedly compared to the in vitro situation. In the glomeruli, which were surrounded by a Bowman’s capsule, a trilaminar GBM, podocyte foot processes, and fenestrated endothelium had appeared. Slit diaphragms between foot processes were starting to form, but were not mature yet. Tubular structures had developed widened lumina with areas displaying an apical brush border [30].

Bantounas et al. generated NPCs from hESCs in 2D culture using a method that was developed by Takasato et al. and entails treatment with CHIR for 3 days followed by FGF9 and heparin for 10 days [32, 86]. On day 12 of differentiation, they dissociated the NPCs and injected them subcutaneously. 12 weeks later, blood vessels lined with human endothelial cells and containing erythrocytes were observed inside and around kidney structures in the grafts and mesangial cells were shown to be present inside the glomerular structures. As a staining for mouse endothelial cells was not performed, it is unclear whether these contributed to organoid vascularization. Similar to the study by van den Berg et al., maturation of transplanted kidney structures was demonstrated using TEM. Upon injection of FITC-labeled dextran into the host, this was found back in some of the organoid tubules, strongly suggesting that glomerular filtration had occurred [32]. However, because glomerular filtration was not directly visualized, it cannot be ruled out that the injected dextran ended up in the tubular structures through a different route. For instance, it could have diffused from neighbouring capillaries into the peritubular space and the tubules.

The vascularization and increased maturation of kidney organoids that occurs upon transplantation improves their resemblance to the in vivo human kidney. However, there are several important limitations to this model.

The vasculature in transplanted organoids is still far from perfect. As discussed previously, the adult human kidney relies on an intricate vascular network lined with endothelial cells that differ in structure and function between kidney compartments for its blood supply and functionality. In the transplanted organoids, fenestrated glomerular endothelial cells resembling those in the adult human kidney have been demonstrated, but the blood vessels outside the glomerular structures have not been extensively characterized. It is, therefore, unclear to what degree their 3D organization and endothelial cells resemble those in vivo. Upon transplantation, the vasculature that is formed does not seem to derive from a main artery, nor does it drain into a main vein. Also, it remains to be elucidated whether the organoid glomerular structures are supplied by one afferent arteriole with their capillary network draining into one efferent arteriole, or by several capillaries that enter the glomeruli at different locations. The second option may result in insufficient glomerular perfusion and on the longer term loss of functionality of the kidney organoids. With regard to the medullary microcirculation, it is unlikely that this is present in the transplanted organoids, considering that their corticomedullary organization is limited and Loops of Henle have not convincingly been demonstrated.

Besides vascularization, a main outcome of these transplantation studies was maturation of structures in transplanted organoids evaluated using TEM imaging. A limitation of TEM analysis is the inability to distinguish between graft and host cells. Theoretically, kidney structures from the mouse host could, therefore, be confused with organoid structures when these are transplanted under the kidney capsule. This risk can be reduced by selecting areas for TEM analysis based on histological sections analyzed with light microscopy, in which organoid and host kidney are easily distinguishable. Inside the transplanted organoids, our experience is that all glomerular structures are of human origin based on stainings for human nuclei. Also, the glomeruli in the mouse kidney are usually not located in the area directly adjacent to the transplanted organoid, further lowering the risk of misidentification.

Another restriction of TEM is its relative subjectiveness. As discussed previously, areas imaged by TEM are selected based on histological sections. This is very useful, but also enables selective analysis. Researchers, therefore, must be careful to image representative regions of the sample and are encouraged to provide a low-magnification histological overview of the sample in addition to the detailed TEM images. An elegant way to reduce cherry picking in TEM imaging is the generation of tile scans, as was done in the study by van den Berg et al., which allows detailed visualization of larger areas.

Of note, in both studies, not all glomerular and tubular structures in the transplanted organoids displayed the same level of maturation and it never reached that of the adult human kidney. In organoids, the glomerular vascular network remains less extensive, the podocyte foot processes and slit diaphragms are not fully mature, glomerular mesangial cells have not been completely characterized, and the apical brush border in tubular structures is not always well developed.

Additional problems that were observed upon in vivo transplantation of kidney organoids are the formation of excessive stromal tissue surrounding the renal structures [30–32] and the lack of a single urinary exit tract.

Despite the limitations discussed above, vascularization and maturation of kidney organoids through transplantation can be useful to improve the modeling of diseases, especially those affecting the podocytes, glomerular endothelial cells or GBM. In a recent study, it was shown using scRNAseq of week 17 human fetal kidneys and kidney organoids generated through the Morizane protocol, that podocytes in organoids follow a similar developmental process as those in human fetal kidneys. However, on day 28 of differentiation, in vitro organoid podocytes are a mix of early and late podocyte like cells, displaying incomplete downregulation of early podocyte genes [28]. Interestingly, they lack expression of several disease-related genes, including COL4A3, which encodes the alpha 3 chain of collagen IV, a major component of the GBM, and is linked to Alport disease. Upon transplantation of days 13–14 organoids under the kidney capsule of NOD SCID mice for 10 days, the organoids were vascularized and did express COL4A3 (IF stainings, not scRNAseq), demonstrating the necessity of vascularization for the normal development of the GBM and consequently for the modeling of Alport disease [28].

The modeling of diseases caused by mutations in genes that are expressed in untransplanted organoids can also be enhanced by vascularization, as was demonstrated in the previously discussed study investigating CNS due to an NPHS1 mutation [16]. In vitro, the authors could detect a difference in NEPHRIN localization between patient and control organoids, but only upon transplantation under the renal capsule of mice and the resulting formation of podocyte foot processes did the impaired development of slit diaphragms in the patient-derived organoids become clear [16]. To enable evaluation of the proteinuric phenotype caused by CNS, functional assessment of kidney organoids, organoid urine production and analysis of this urine will be necessary which, to our knowledge, has not been achieved yet.

Although vascularization of kidney organoids can improve disease modeling, it also complicates experiments. It will, therefore, depend on the research question and on the disease being studied if vascularized organoids are the most suitable model.

### In vitro vascularization of hPSC-derived kidney organoids

Recently, Homan et al. [33] aimed to generate kidney organoids containing a perfusable vascular network in vitro by culturing them under flow. They differentiated PSCs to kidney organoids using the protocol developed by Morizane et al. [6] and on day 11 of differentiation placed them on perfusable millifluidic chips, on which the medium flows over the top of the organoids. This induced the expansion of the organoid-derived endothelial cell network that, in some cases, entered the glomerular structures and aligned the tubuli. The authors claimed that some of the blood vessels were perfusable based on the apparent overlap of fluorescent beads which were added to the media with endothelial cells stained with *Ulex europaeus* lectin or CD31 [33]. Due to the diffuse presence of the fluorescent beads in the image and video published by the authors and the small number of vessels for which the overlap was shown, this claim is not irrefutable. Transmission electron microscopy imaging of the organoids cultured under flow demonstrated the appearance of a Bowman’s capsule like structure and improved maturation of podocyte foot processes. A glomerular basement membrane was not demonstrated in the published TEM images of organoid glomeruli, indicating that the interaction between the endothelial cells and podocytes was not optimal.

Neither the vascular network nor the maturation in this model was therefore as extensive as those observed after transplantation. There was, however, an expansion of the endothelial cell network which invaded some of the organoid glomerular structures. The addition of flow to the system seems to have been an important factor causing this, although the large changes that were made to the extracellular matrix and the addition of fetal bovine serum to the medium likely contributed as well.

In summary, in vivo transplantation of kidney organoids leads to a certain degree of vascularization and maturation, which can improve the modeling of glomerular kidney disease and glomerulotoxicity and is an important step towards using kidney organoids for regenerative medicine. However, the 3D organization of the vasculature as well as the nephrons in transplanted kidney organoids are not as advanced as that in the adult human kidney. In vitro, functional perfusion of vascularized kidney organoids has yet to be achieved.

## Conclusions and future perspectives

Since the discovery of iPSCs, great progress has been made in the field of kidney organoid generation. Robust protocols have been developed for the differentiation of PSCs to segmented nephrons and adapting culture conditions enables large-scale production. In their current form, organoids are useful to study the early developmental processes, model certain early onset kidney diseases, and screen for tubular nephrotoxicity. Transplantation of kidney organoids in murine hosts or in vitro culture under flow can be performed to accomplish a certain degree of vascularization and maturation, increasing the applicability of the organoids. Nonetheless, we still have a long way to go towards generating fully functional kidney tissue with a specialized vasculature and organized urine drainage system. Also, before kidney organoids can be introduced for regenerative medicine applications, pressing safety issues must be addressed. Organoids are subject to interexperimental variability, frequently contain off-target cell populations, and upon transplantation, avoiding immunological rejection is a great challenge.

For the future, we expect that achieving functional vascularization of kidney organoids in vitro combined with advancements increasing their safety will markedly enhance their potential.

## References

[1] Matsushita K, van der Velde M, Astor BC, Woodward M, Levey AS, de Jong PE, Coresh J, Gansevoort RT (2010). Association of estimated glomerular filtration rate and albuminuria with all-cause and cardiovascular mortality in general population cohorts: a collaborative meta-analysis. Lancet.

[2] Go AS, Chertow GM, Fan D, McCulloch CE, Hsu CY (2004). Chronic kidney disease and the risks of death, cardiovascular events, and hospitalization. N Engl J Med.

[3] van der Velde M, Matsushita K, Coresh J, Astor BC, Woodward M, Levey A, de Jong P, Gansevoort RT, Chronic Kidney Disease Prognosis C (2011). Lower estimated glomerular filtration rate and higher albuminuria are associated with all-cause and cardiovascular mortality. A collaborative meta-analysis of high-risk population cohorts. Kidney Int.

[4] Takahashi K, Tanabe K, Ohnuki M, Narita M, Ichisaka T, Tomoda K, Yamanaka S (2007). Induction of pluripotent stem cells from adult human fibroblasts by defined factors. Cell.

[5] Taguchi A, Kaku Y, Ohmori T, Sharmin S, Ogawa M, Sasaki H, Nishinakamura R (2014). Redefining the in vivo origin of metanephric nephron progenitors enables generation of complex kidney structures from pluripotent stem cells. Cell Stem Cell.

[6] Morizane R, Lam AQ, Freedman BS, Kishi S, Valerius MT, Bonventre JV (2015). Nephron organoids derived from human pluripotent stem cells model kidney development and injury. Nat Biotechnol.

[7] Takasato M, Er PX, Chiu HS, Maier B, Baillie GJ, Ferguson C, Parton RG, Wolvetang EJ, Roost MS, de Sousa Chuva, Lopes SM, Little MH (2015). Kidney organoids from human iPS cells contain multiple lineages and model human nephrogenesis. Nature.

[8] Freedman BS, Brooks CR, Lam AQ, Fu H, Morizane R, Agrawal V, Saad AF, Li MK, Hughes MR, Werff RV, Peters DT, Lu J, Baccei A, Siedlecki AM, Valerius MT, Musunuru K, McNagny KM, Steinman TI, Zhou J, Lerou PH, Bonventre JV (2015). Modelling kidney disease with CRISPR-mutant kidney organoids derived from human pluripotent epiblast spheroids. Nat Commun.

[9] Boyle S, Misfeldt A, Chandler KJ, Deal KK, Southard-Smith EM, Mortlock DP, Baldwin HS, de Caestecker M (2008). Fate mapping using Cited1-CreERT2 mice demonstrates that the cap mesenchyme contains self-renewing progenitor cells and gives rise exclusively to nephronic epithelia. Dev Biol.

[10] Herzlinger D, Koseki C, Mikawa T, al-Awqati Q (1992). Metanephric mesenchyme contains multipotent stem cells whose fate is restricted after induction. Development.

[11] Kobayashi A, Valerius MT, Mugford JW, Carroll TJ, Self M, Oliver G, McMahon AP (2008). Six2 defines and regulates a multipotent self-renewing nephron progenitor population throughout mammalian kidney development. Cell Stem Cell.

[12] Grobstein C (1955). Inductive interaction in the development of the mouse metanephros. J Exp Zool.

[13] Auerbach R, Grobstein C (1958). Inductive interaction of embryonic tissues after dissociation and reaggregation. Exp Cell Res.

[14] Taguchi A, Nishinakamura R (2017). Higher-order kidney organogenesis from pluripotent stem cells. Cell Stem Cell.

[15] Kim YK, Refaeli I, Brooks CR, Jing P, Gulieva RE, Hughes MR, Cruz NM, Liu Y, Churchill AJ, Wang Y, Fu H, Pippin JW, Lin LY, Shankland SJ, Vogl AW, McNagny KM, Freedman BS (2017). Gene-edited human kidney organoids reveal mechanisms of disease in podocyte development. Stem Cells.

[16] Tanigawa S, Islam M, Sharmin S, Naganuma H, Yoshimura Y, Haque F, Era T, Nakazato H, Nakanishi K, Sakuma T, Yamamoto T, Kurihara H, Taguchi A, Nishinakamura R (2018). Organoids from nephrotic disease-derived iPSCs identify impaired NEPHRIN localization and slit diaphragm formation in kidney podocytes. Stem Cell Rep.

[17] Forbes TA, Howden SE, Lawlor K, Phipson B, Maksimovic J, Hale L, Wilson S, Quinlan C, Ho G, Holman K, Bennetts B, Crawford J, Trnka P, Oshlack A, Patel C, Mallett A, Simons C, Little MH (2018). Patient-iPSC-derived kidney organoids show functional validation of a ciliopathic renal phenotype and reveal underlying pathogenetic mechanisms. Am J Hum Genet.

[18] Hale LJ, Howden SE, Phipson B, Lonsdale A, Er PX, Ghobrial I, Hosawi S, Wilson S, Lawlor KT, Khan S, Oshlack A, Quinlan C, Lennon R, Little MH (2018). 3D organoid-derived human glomeruli for personalised podocyte disease modelling and drug screening. Nat Commun.

[19] Cruz NM, Song X, Czerniecki SM, Gulieva RE, Churchill AJ, Kim YK, Winston K, Tran LM, Diaz MA, Fu H, Finn LS, Pei Y, Himmelfarb J, Freedman BS (2017). Organoid cystogenesis reveals a critical role of microenvironment in human polycystic kidney disease. Nat Mater.

[20] Wu H, Uchimura K, Donnelly EL, Kirita Y, Morris SA, Humphreys BD (2018). Comparative analysis and refinement of human PSC-derived kidney organoid differentiation with single-cell transcriptomics. Cell Stem Cell.

[21] Phipson B, Er PX, Combes AN, Forbes TA, Howden SE, Zappia L, Yen HJ, Lawlor KT, Hale LJ, Sun J, Wolvetang E, Takasato M, Oshlack A, Little MH (2019). Evaluation of variability in human kidney organoids. Nat Methods.

[22] Czerniecki SM, Cruz NM, Harder JL, Menon R, Annis J, Otto EA, Gulieva RE, Islas LV, Kim YK, Tran LM, Martins TJ, Pippin JW, Fu H, Kretzler M, Shankland SJ, Himmelfarb J, Moon RT, Paragas N, Freedman BS (2018). High-throughput screening enhances kidney organoid differentiation from human pluripotent stem cells and enables automated multidimensional phenotyping. Cell Stem Cell.

[23] Combes AN, Zappia L, Er PX, Oshlack A, Little MH (2019). Single-cell analysis reveals congruence between kidney organoids and human fetal kidney. Genome Med.

[24] Gornalusse GG, Hirata RK, Funk SE, Riolobos L, Lopes VS, Manske G, Prunkard D, Colunga AG, Hanafi LA, Clegg DO, Turtle C, Russell DW (2017). HLA-E-expressing pluripotent stem cells escape allogeneic responses and lysis by NK cells. Nat Biotechnol.

[25] Sasaki H, Wada H, Baghdadi M, Tsuji H, Otsuka R, Morita K, Shinohara N, Seino K (2015). New immunosuppressive cell therapy to prolong survival of induced pluripotent stem cell-derived allografts. Transplantation.

[26] Eremina V, Sood M, Haigh J, Nagy A, Lajoie G, Ferrara N, Gerber HP, Kikkawa Y, Miner JH, Quaggin SE (2003). Glomerular-specific alterations of VEGF-A expression lead to distinct congenital and acquired renal diseases. J Clin Invest.

[27] Lindahl P, Hellstrom M, Kalen M, Karlsson L, Pekny M, Pekna M, Soriano P, Betsholtz C (1998). Paracrine PDGF-B/PDGF-Rbeta signaling controls mesangial cell development in kidney glomeruli. Development.

[28] Tran T, Lindstrom NO, Ransick A, Brandine GD, Guo QY, Kim AD, Der B, Peti-Peterdi J, Smith AD, Thornton M, Grubbs B, McMahon JA, McMahon AP (2019). In vivo developmental trajectories of human podocyte inform in vitro differentiation of pluripotent stem cell-derived podocytes. Dev Cell.

[29] Kenig-Kozlovsky Y, Scott RP, Onay T, Carota IA, Thomson BR, Gil HJ, Ramirez V, Yamaguchi S, Tanna CE, Heinen S, Wu C, Stan RV, Klein JD, Sands JM, Oliver G, Quaggin SE (2018). Ascending vasa recta are angiopoietin/Tie2-dependent lymphatic-like vessels. J Am Soc Nephrol.

[30] van den Berg CW, Ritsma L, Avramut MC, Wiersma LE, van den Berg BM, Leuning DG, Lievers E, Koning M, Vanslambrouck JM, Koster AJ, Howden SE, Takasato M, Little MH, Rabelink TJ (2018). Renal subcapsular transplantation of PSC-derived kidney organoids induces neo-vasculogenesis and significant glomerular and tubular maturation in vivo. Stem Cell Rep.

[31] Sharmin S, Taguchi A, Kaku Y, Yoshimura Y, Ohmori T, Sakuma T, Mukoyama M, Yamamoto T, Kurihara H, Nishinakamura R (2016). Human induced pluripotent stem cell-derived podocytes mature into vascularized glomeruli upon experimental transplantation. J Am Soc Nephrol.

[32] Bantounas I, Ranjzad P, Tengku F, Silajdzic E, Forster D, Asselin MC, Lewis P, Lennon R, Plagge A, Wang Q, Woolf AS, Kimber SJ (2018). Generation of functioning nephrons by implanting human pluripotent stem cell-derived kidney progenitors. Stem Cell Rep.

[33] Homan KA, Gupta N, Kroll KT, Kolesky DB, Skylar-Scott M, Miyoshi T, Mau D, Valerius MT, Ferrante T, Bonventre JV, Lewis JA, Morizane R (2019). Flow-enhanced vascularization and maturation of kidney organoids in vitro. Nat Methods.

[34] Mugford JW, Sipila P, McMahon JA, McMahon AP (2008). Osr1 expression demarcates a multi-potent population of intermediate mesoderm that undergoes progressive restriction to an Osr1-dependent nephron progenitor compartment within the mammalian kidney. Dev Biol.

[35] Saxen L, Sariola H (1987). Early organogenesis of the kidney. Pediatr Nephrol.

[36] Moore MW, Klein RD, Farinas I, Sauer H, Armanini M, Phillips H, Reichardt LF, Ryan AM, Carver-Moore K, Rosenthal A (1996). Renal and neuronal abnormalities in mice lacking GDNF. Nature.

[37] Pichel JG, Shen L, Sheng HZ, Granholm AC, Drago J, Grinberg A, Lee EJ, Huang SP, Saarma M, Hoffer BJ, Sariola H, Westphal H (1996). Defects in enteric innervation and kidney development in mice lacking GDNF. Nature.

[38] Sanchez MP, Silos-Santiago I, Frisen J, He B, Lira SA, Barbacid M (1996). Renal agenesis and the absence of enteric neurons in mice lacking GDNF. Nature.

[39] Carroll TJ, Park JS, Hayashi S, Majumdar A, McMahon AP (2005). Wnt9b plays a central role in the regulation of mesenchymal to epithelial transitions underlying organogenesis of the mammalian urogenital system. Dev Cell.

[40] Karner CM, Das A, Ma Z, Self M, Chen C, Lum L, Oliver G, Carroll TJ (2011). Canonical Wnt9b signaling balances progenitor cell expansion and differentiation during kidney development. Development.

[41] Tanigawa S, Wang H, Yang Y, Sharma N, Tarasova N, Ajima R, Yamaguchi TP, Rodriguez LG, Perantoni AO (2011). Wnt4 induces nephronic tubules in metanephric mesenchyme by a non-canonical mechanism. Dev Biol.

[42] Burn SF, Webb A, Berry RL, Davies JA, Ferrer-Vaquer A, Hadjantonakis AK, Hastie ND, Hohenstein P (2011). Calcium/NFAT signalling promotes early nephrogenesis. Dev Biol.

[43] Lindstrom NO (2018). Progressive recruitment of mesenchymal progenitors reveals a time-dependent process of cell fate acquisition in mouse and human nephrogenesis. Dev Cell.

[44] Combes AN, Phipson B, Lawlor KT, Dorison A, Patrick R, Zappia L, Harvey RP, Oshlack A, Little MH (2019). Single cell analysis of the developing mouse kidney provides deeper insight into marker gene expression and ligand-receptor crosstalk. Development.

[45] Kaku Y, Taguchi A, Tanigawa S, Haque F, Sakuma T, Yamamoto T, Nishinakamura R (2017). PAX2 is dispensable for in vitro nephron formation from human induced pluripotent stem cells. Sci Rep.

[46] Combes AN, Phipson B, Zappia L, Lawlor K, Er PX, Oshlack A, Little MH (2017) High throughput single cell RNA-seq of developing mouse kidney and human kidney organoids reveals a roadmap for recreating the kidney. bioRxiv Preprint

[47] Unbekandt M, Davies JA (2010). Dissociation of embryonic kidneys followed by reaggregation allows the formation of renal tissues. Kidney Int.

[48] Ganeva V, Unbekandt M, Davies JA (2011). An improved kidney dissociation and reaggregation culture system results in nephrons arranged organotypically around a single collecting duct system. Organogenesis.

[49] Przepiorski A, Sander V, Tran T, Hollywood JA, Sorrenson B, Shih JH, Wolvetang EJ, McMahon AP, Holm TM, Davidson AJ (2018). A simple bioreactor-based method to generate kidney organoids from pluripotent stem cells. Stem Cell Rep.

[50] Boulting GL, Kiskinis E, Croft GF, Amoroso MW, Oakley DH, Wainger BJ, Williams DJ, Kahler DJ, Yamaki M, Davidow L, Rodolfa CT, Dimos JT, Mikkilineni S, MacDermott AB, Woolf CJ, Henderson CE, Wichterle H, Eggan K (2011). A functionally characterized test set of human induced pluripotent stem cells. Nat Biotechnol.

[51] Bock C, Kiskinis E, Verstappen G, Gu H, Boulting G, Smith ZD, Ziller M, Croft GF, Amoroso MW, Oakley DH, Gnirke A, Eggan K, Meissner A (2011). Reference Maps of human ES and iPS cell variation enable high-throughput characterization of pluripotent cell lines. Cell.

[52] Fu Y, Foden JA, Khayter C, Maeder ML, Reyon D, Joung JK, Sander JD (2013). High-frequency off-target mutagenesis induced by CRISPR–Cas nucleases in human cells. Nat Biotechnol.

[53] Hsu PD, Scott DA, Weinstein JA, Ran FA, Konermann S, Agarwala V, Li Y, Fine EJ, Wu X, Shalem O, Cradick TJ, Marraffini LA, Bao G, Zhang F (2013). DNA targeting specificity of RNA-guided Cas9 nucleases. Nat Biotechnol.

[54] Lin Y, Cradick TJ, Brown MT, Deshmukh H, Ranjan P, Sarode N, Wile BM, Vertino PM, Stewart FJ, Bao G (2014). CRISPR/Cas9 systems have off-target activity with insertions or deletions between target DNA and guide RNA sequences. Nucleic Acids Res.

[55] Moffat DB, Fourman J (2001). A vascular pattern of the rat kidney. 1963. J Am Soc Nephrol.

[56] Eremina V, Cui S, Gerber H, Ferrara N, Haigh J, Nagy A, Ema M, Rossant J, Jothy S, Miner JH, Quaggin SE (2006). Vascular endothelial growth factor a signaling in the podocyte-endothelial compartment is required for mesangial cell migration and survival. J Am Soc Nephrol.

[57] Bearer EL, Orci L (1985). Endothelial fenestral diaphragms: a quick-freeze, deep-etch study. J Cell Biol.

[58] Beeuwkes R (1971). Efferent vascular patterns and early vascular-tubular relations in the dog kidney. Am J Physiol.

[59] Breier G, Albrecht U, Sterrer S, Risau W (1992). Expression of vascular endothelial growth factor during embryonic angiogenesis and endothelial cell differentiation. Development.

[60] Bartlett CS, Jeansson M, Quaggin SE (2016). Vascular growth factors and glomerular disease. Annu Rev Physiol.

[61] Fierlbeck W, Liu A, Coyle R, Ballermann BJ (2003). Endothelial cell apoptosis during glomerular capillary lumen formation in vivo. J Am Soc Nephrol.

[62] Ichimura K, Stan RV, Kurihara H, Sakai T (2008). Glomerular endothelial cells form diaphragms during development and pathologic conditions. J Am Soc Nephrol.

[63] Reeves WH, Kanwar YS, Farquhar MG (1980). Assembly of the glomerular filtration surface. Differentiation of anionic sites in glomerular capillaries of newborn rat kidney. J Cell Biol.

[64] Kitamoto Y, Tokunaga H, Tomita K (1997). Vascular endothelial growth factor is an essential molecule for mouse kidney development: glomerulogenesis and nephrogenesis. J Clin Invest.

[65] Sison K, Eremina V, Baelde H, Min W, Hirashima M, Fantus IG, Quaggin SE (2010). Glomerular structure and function require paracrine, not autocrine, VEGF-VEGFR-2 signaling. J Am Soc Nephrol.

[66] Leveen P, Pekny M, Gebre-Medhin S, Swolin B, Larsson E, Betsholtz C (1994). Mice deficient for PDGF B show renal, cardiovascular, and hematological abnormalities. Genes Dev.

[67] Soriano P (1994). Abnormal kidney development and hematological disorders in PDGF beta-receptor mutant mice. Genes Dev.

[68] Risau W (1997). Mechanisms of angiogenesis. Nature.

[69] Carmeliet P (2000). Mechanisms of angiogenesis and arteriogenesis. Nat Med.

[70] Robert B, St John PL, Abrahamson DR (1998). Direct visualization of renal vascular morphogenesis in Flk1 heterozygous mutant mice. Am J Physiol.

[71] Loughna S, Hardman P, Landels E, Jussila L, Alitalo K, Woolf AS (1997). A molecular and genetic analysis of renalglomerular capillary development. Angiogenesis.

[72] Sims-Lucas S, Schaefer C, Bushnell D, Ho J, Logar A, Prochownik E, Gittes G, Bates CM (2013). Endothelial progenitors exist within the kidney and lung mesenchyme. PLoS ONE.

[73] Hyink DP, Tucker DC, St John PL, Leardkamolkarn V, Accavitti MA, Abrass CK, Abrahamson DR (1996). Endogenous origin of glomerular endothelial and mesangial cells in grafts of embryonic kidneys. Am J Physiol.

[74] Robert B, St John PL, Hyink DP, Abrahamson DR (1996). Evidence that embryonic kidney cells expressing flk-1 are intrinsic, vasculogenic angioblasts. Am J Physiol.

[75] Rogers SA, Hammerman MR (2001). Transplantation of rat metanephroi into mice. Am J Physiol Regul Integr Comp Physiol.

[76] Takeda S, Rogers SA, Hammerman MR (2006). Differential origin for endothelial and mesangial cells after transplantation of pig fetal renal primordia into rats. Transpl Immunol.

[77] Dekel B, Burakova T, Arditti FD, Reich-Zeliger S, Milstein O, Aviel-Ronen S, Rechavi G, Friedman N, Kaminski N, Passwell JH, Reisner Y (2003). Human and porcine early kidney precursors as a new source for transplantation. Nat Med.

[78] Munro DAD, Hohenstein P, Davies JA (2017). Cycles of vascular plexus formation within the nephrogenic zone of the developing mouse kidney. Sci Rep.

[79] Rogers SA, Lowell JA, Hammerman NA, Hammerman MR (1998). Transplantation of developing metanephroi into adult rats. Kidney Int.

[80] Rogers SA, Talcott M, Hammerman MR (2003). Transplantation of pig metanephroi. ASAIO J.

[81] Dekel B, Amariglio N, Kaminski N, Schwartz A, Goshen E, Arditti FD, Tsarfaty I, Passwell JH, Reisner Y, Rechavi G (2002). Engraftment and differentiation of human metanephroi into functional mature nephrons after transplantation into mice is accompanied by a profile of gene expression similar to normal human kidney development. J Am Soc Nephrol.

[82] Dilworth MR, Clancy MJ, Marshall D, Bravery CA, Brenchley PE, Ashton N (2008). Development and functional capacity of transplanted rat metanephroi. Nephrol Dial Transplant.

[83] Rogers SA, Liapis H, Hammerman MR (2001). Transplantation of metanephroi across the major histocompatibility complex in rats. Am J Physiol Regul Integr Comp Physiol.

[84] Xinaris C, Benedetti V, Rizzo P, Abbate M, Corna D, Azzollini N, Conti S, Unbekandt M, Davies JA, Morigi M, Benigni A, Remuzzi G (2012). In vivo maturation of functional renal organoids formed from embryonic cell suspensions. J Am Soc Nephrol.

[85] Takebe T, Enomura M, Yoshizawa E, Kimura M, Koike H, Ueno Y, Matsuzaki T, Yamazaki T, Toyohara T, Osafune K, Nakauchi H, Yoshikawa HY, Taniguchi H (2015). Vascularized and complex organ buds from diverse tissues via mesenchymal cell-driven condensation. Cell Stem Cell.

[86] Takasato M, Er PX, Becroft M, Vanslambrouck JM, Stanley EG, Elefanty AG, Little MH (2014). Directing human embryonic stem cell differentiation towards a renal lineage generates a self-organizing kidney. Nat Cell Biol.

